# The use of intrathecal morphine for acute postoperative pain in lower limb arthroplasty surgery: a survey of practice at an academic hospital

**DOI:** 10.1186/s13018-022-03215-0

**Published:** 2022-06-21

**Authors:** Mpumelelo Sibanyoni, Ntombiyethu Biyase, Palesa Motshabi Chakane

**Affiliations:** Department of Anaesthesiology, University of the Witwatersrand, Johannesburg, South Africa

**Keywords:** Intrathecal morphine, Spinal anaesthesia, Hip arthroplasty, Knee arthroplasty

## Abstract

**Background and purpose of the study:**

Intrathecal morphine (ITM) provides optimal postoperative analgesia in patients who are scheduled for total knee and hip operation with spinal anaesthesia. However, the ideal dose at which maximal analgesic effect occurs with minimal side effects is not known. This study aimed to describe the use of two doses of ITM and side effect profile in patients undergoing elective hip and knee arthroplasty.

**Methods:**

This was a prospective, descriptive, and contextual study conducted on patients who had total hip and knee replacement at Chris Hani Baragwanath Academic Hospital from 1 September to 30 November 2020. The sample size consisted of 66 patients who were 18 years and older, American Society of Anaesthesiology (ASA) classification 1–3, patients who had received either 100 mcg or 150 mcg ITM dose under spinal anaesthesia and sent to the ward postoperatively. Visual Analogue Scale (VAS) score was used to assess pain in the first 24 h, consumption of rescue analgesia and reported side effects were documented.

**Results:**

There was no relationship between age, weight, ASA classification or type of surgery and VAS score classification groups. Patients who received 100 mcg ITM had a higher median VAS pain score 2 (1–5) compared to those who received 150 mcg ITM 1 (0–2), *p* = 0.01. The need for rescue analgesia between the two groups was marginally less in the 150 mcg ITM group (*p* = 0.098). There was no difference in the rate of side effects between the 100 mcg ITM group [12 (41%)] and the 150 mcg ITM group [17 (59%)], *p* = 0.92. Rescue analgesia was marginally different between groups, *p* = 0.09. There were no real differences in the VAS pain scores between the total knee and total hip surgeries. None of the patients experienced clinically significant respiratory depression.

**Conclusion:**

The 150 mcg ITM dose provided good analgesic effects with longer duration of action and comparable side effect profile to the 100 mcg ITM dose. This dose was not associated with development of respiratory depression and can therefore be administered safely to patients who are discharged to the ward postoperatively in a resource constraint environment.

## Background

In 1979, Wang demonstrated the efficacy of intrathecal morphine (ITM) for postoperative analgesia in patients with genitourinary cancer; since then, ITM has become widely used [1]. Morphine was the first opioid to be approved by Food and Drug Administration for its use in neuraxial anaesthesia [1, 2]. The added benefits of using ITM are that it is simple to administer, it is not associated with increase in blood loss during surgery and has longer duration of action [3]. It is cost effective as it leads to early mobilization and decrease hospital stay [2, 4, 5].

Morphine is hydrophilic and binds with high affinity to the central nervous system opioids receptors. This results in its prolonged presence within the water-soluble cerebrospinal fluid resulting in prolonged duration of action up to 24 h and its cause of delayed respiratory depression [1, 6, 7]. Intrathecal morphine also causes other side effects such as postoperative nausea and vomiting, pruritus, and urinary retention [1, 8]. Long duration of analgesia has made ITM a suitable option for postoperative analgesia in patients who are scheduled for total knee and hip operation with spinal anaesthesia [3].

The international consensus on anaesthesia-related outcome after surgery group has recommended the use of neuraxial anaesthesia during joint replacement [9]. Many studies have endeavoured to define or determine the low dose of ITM with excellent analgesic effects and minimal or no side effects [8]. The dose of 100–200 mcg ITM has been shown to provide a good balance between analgesia and side effects [10, 11]. Most patients that are scheduled for hip and knee arthroplasty are elderly; intrathecal morphine has the advantage of reducing the stress response and metabolic demand during surgery and anaesthesia in the elderly [12]. There is contradicting evidence of where patients should be recovered post-surgery with ITM [1, 10].

We sought to assess the analgesic effect of two doses of ITM, describe the perioperative outcomes ITM-related complications/side effects and to assess the safety of managing these patients in a normal ward in a resource constraint environment of arguably the largest hospital on the continent, Chris Hani Baragwanath Academic Hospital [13].

## Methods

The study was conducted in the Arthroplasty unit and the Department of Anaesthesiology at Chris Hani Baragwanath Academic Hospital, affiliated with the Faculty of Health Sciences of the University of the Witwatersrand. Approval to conduct the study was obtained from the Human Research Ethics Committee and the University of the Witwatersrand and other relevant authorities. The study followed a prospective, descriptive, and contextual research design. This study aimed to describe the use of ITM for perioperative pain in patients undergoing hip and knee arthroplasty who were discharged to the ward postoperatively and the side effect profile associated with ITM injection use.

The study population included patients 18 years and older, American Society of Anaesthesiology (ASA) physical status 1-3 scheduled for elective knee and hip arthroplasty who received ITM injection and spinal anaesthesia and sent to the ward postoperatively. Written informed consent was obtained from all patients included in the study. Patients were excluded if they were having obstructive sleep apnoea, respiratory diseases, ASA physical status 4 and above, had receive general anaesthetic and single shot intrathecal morphine, and patient records which were illegible. The study period was from 1 September to 30 November 2020. After the procedure, patients were monitored in the recovery room then discharged to the ward with satisfactory vital signs including blood pressure and saturation. In the ward patients were monitored for postoperative pain, pruritus, nausea and vomiting and respiratory depression for the first 24 h. Respiratory depression was defined as respiratory rate of less than 10 breaths per minutes or oxygen saturation of less than 90%. Patients were treated for the side effects if they experience any. Data on rehabilitation including continuous passive motion, and constipation of the patients were not captured for this study.

Pain scores were evaluated using a Visual Analogue Scale from 0 to 10 with 0 indicating no pain and 10 severe pain. Rescue analgesia and time from ITM administration were also documented. The rescue analgesia regimen used was standard management in the orthopaedic ward consisting of paracetamol 1 g, tramadol hydrochloride dose 50–100 mg and pethidine 50–100 mg and administered 8-hourly when necessary (PRN). The pain-free hours were divided into 3-h periods, and the median VAS pain scores within the first 24 h were determined for each group.

## Statistical analysis

Sixty-six patients participated in the study. The categorical variables were described using frequencies and percentages. We used the Chi-square test for significance of association between categorical variables and the intrathecal morphine dose (outcome). The Fisher’s exact test was used to find the association between categorical variables (age, ASA and type of surgery) and VAS pain scores. Continuous variables were described using median and interquartile ranges since they were not normally distributed according to the Shapiro–Wilk test. The relationship between continuous variables and intrathecal morphine dose (outcome) was assessed using the Mann–Whitney test. Weight and VAS pain scores association was assessed using the Kruskal–Wallis test which is a nonparametric alternative to ANOVA.

## Results

Sixty-six patients received spinal anaesthesia with ITM injection. Of the 66 patients, 29 (44%) received 100 mcg ITM dose and 37 (56%) received 150 mcg ITM dose. From the 100 mcg ITM dose group, 11 (38%) were male and 18 (62%) were female (Table 1). From the 150 mcg ITM dose group, 19 (51%) were male and 18 (49%) were female. Majority of the patients were 50 years old and older. The median (IQR) weight of the patients was 75 (65–79) kg in the 100 mcg ITM group and 73 (65–80) kg in the 150 mcg ITM group (Table 1).

**Table 1:** Demographic and baseline characteristics

Variable		Overall *n* = 66	100 mcg ITM *n* = 29	150 mcg ITM *n* = 37	*P*-values
Gender	Male	30 (45)	11 (37)	19 (63)	0.28
	Female	36 (55)	18 (50)	18 (50)	
Age in years	< 40	7 (11)	0 (0.0)	7 (100)	0.012
	40–49	5 (8)	1 (20)	4 (80)	
	50–59	19 (29)	10 (53)	9 (47)	
	60–69	23 (31)	9 (39)	14 (61)	
	70+	12 (18)	9 (75)	3 (25)	
Weight [median (IQR)]		74.5 (65–80)	75 (65–79)	73 (65–80)	0.81
ASA	I	8 (12)	3 (38)	5 (62)	0.54
	II	53 (80)	25 (47)	28 (53)	
	III	5 (7.58)	1 (20)	4 (80)	
Surgery	THR	48 (73)	23 (48)	25 (52)	0.29
	TKR	18 (27)	6 (33)	12 (67)	
Bupivacaine	Median (IQR)	14 (13–14.5)	13 (12–14)	14 (14–15)	< 0.001

*ITM* intrathecal morphine, *THR* total hip replacement, *TKR* total knee replacement, *ASA* American Society of Anaesthesiology

In total, 8 (12.12%) patients were classified as ASA I, 53 (80%) ASA II and 5 (8%) were ASA III. There were 48 (73%) who underwent hip arthroplasty patients compared to 18 (27%) who were underwent knee arthroplasty in the study. Within the hip arthroplasty group, 23 (48%) had 100 mcg IT morphine dose, while only 6 (33%) of the knee arthroplasty received 100 mcg IT morphine. Twenty-five (52%) patients received 150 mcg IT morphine from the hip arthroplasty group and 12 (67%) patients from the knee arthroplasty group. The overall bupivacaine dose administered was median (IQR) was 14 (13–14.5), 13 (12–14) in the 100 mcg ITM group and 14 (14–15) in the 150 mcg ITM group.

There was no relationship between age, weight, ASA classification or type of surgery between VAS score classification groups (Table 2).

**Table 2:** Comparison between VAS pain scores and basic characteristics categories

Variable		VAS pain scores *n* (%)				*p*-value
		None	Mild 1–3	Moderate 4–6	Severe > 6	
Age in years	< 40	3 (43)	2 (29)	1 (14)	1 (14)	0.45
	40–49	2 (40)	1 (20)	2 (40)	0 (0)	
	50–59	4 (21)	10 (53)	4 (21)	1 (5)	
	60–69	4 (17)	14 (61)	1 (4)	4 (18)	
	70+	2 (17)	6 (50)	2 (17)	2 (17)	
Weight	Median (IQR)	73 (60–85)	70 (65–80)	73 (69–79)	75 (75–81)	0.48
ASA	I	1 (12)	5 (63)	1 (12.5)	1 (12.5)	0.61
	II	13 (25)	26 (49)	9 (17)	5 (9)	
	III	1 (20)	2 (40)	0 (0)	2 (40)	
Surgery	THR	11 (23)	24 (50)	9 (19)	4 (8)	0.34
	TKR	4 (22)	9 (50)	1 (6)	4 (22)	

*VAS* Visual Analogue Scale

A total of 29 (44%) patients did not have side effects in the study. There was no difference in the rate of side effects between the 100 mcg ITM group [12 (41%)] and the 150 mcg ITM group [17 (59%)], *p* = 0.92 (Table 3). Pruritus was not dose dependant as 11 (50%) patients in each ITM group had pruritus with a total of 22 (33%) overall. The overall number of patients who developed nausea and vomiting was 5 (8%) with 2 (40%) patients in the 100 mcg ITM group and 3 (60%) in the 150 mcg ITM group. Patients who had both pruritus, nausea and vomiting accounted for only 10 (15%) patients of which 4 (40%) were in the 100 mcg ITM group and 6 (60%) in the 150 mcg ITM group. None of the patients experienced clinically significant respiratory depression.

**Table 3:** Comparison of postoperative outcomes by ITM dose

Variable		Overall *n* = 66	100 mcg ITM *n* = 29	150 mcg ITM *n* = 37	*p*-value
Side effects	None	29 (44)	12 (41)	17 (59)	0.92
	Pruritus	22 (33)	11 (50)	11 (50)	
	Nausea & vomiting	5 (8)	2 (40)	3 (60)	
	Both	10 (15)	4 (40)	6 (60)	
PEP	Yes	21 (32)	10 (48)	11 (53)	0.68
	No	45 (68)	19 (42)	26 (58)	
Ephedrine	Yes	7 (11)	4 (57)	3 (43)	0.46
	No	59 (89)	25 (42)	34 (58)	
Rescue analgesia	Paracetamol	3 (5)	3 (100)	0 (0)	0.098
	Tramadol hydrochloride	21 (32)	11 (52)	10 (48)	
	Pethidine	15 (23)	5 (33)	10 (67)	
	Paracetamol & tramadol hydrochloride	6 (9)	4 (45)	2 (33)	
	Paracetamol & pethidine	2 (3)	1 (50)	1 (50)	
	None	19 (29)	5 (26)	14 (74)	
Pain-free time (min) [median (IQR)]		720 (540–1110)	675 (540–785)	720 (550–1440)	0.31
VAS score median (IQR)		2 (1–4)	2 (1–5)	1 (0–2)	0.01

*VAS* Visual Analogue Scale, *PEP* phenylephrine, *ITM* intrathecal morphine

There was a significant difference in median (IQR) VAS pain scores between the 100 mcg ITM and the 150 mcg ITM groups [2 (1–5) and 1 (0–2), respectively], *p* = 0.01. The need for rescue analgesia between the 100 and 150 mcg ITM groups was marginally different (*p* = 0.098). Of note is that 19 (29%) patients did not require rescue analgesia. The use of phenylephrine (PEP) and ephedrine was not different between groups.

**Fig. 1:**
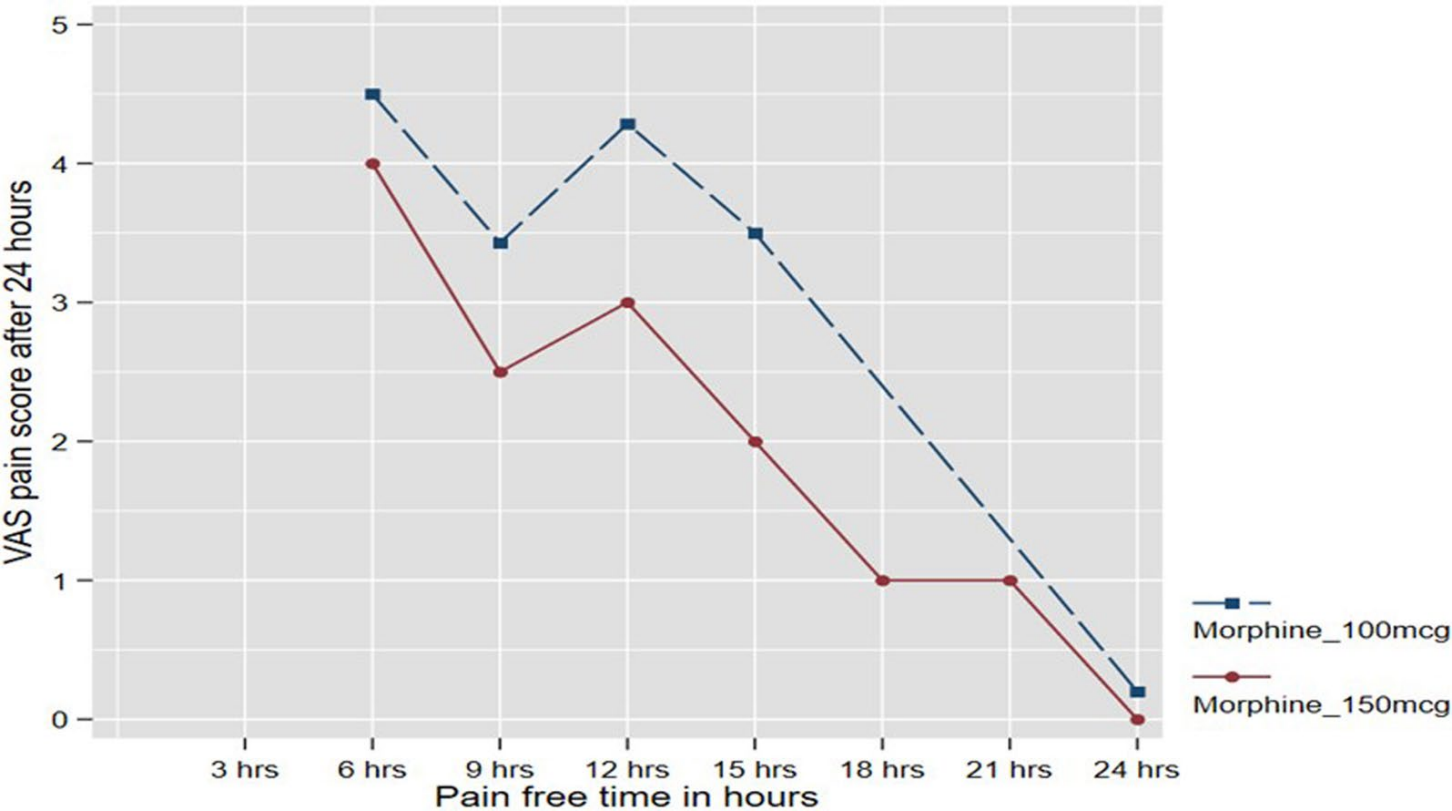
VAS pain scores for patients given 100 mcg ITM group and the 150 mcg ITM group adjusted for the number of hours it took for the patient to start feeling pain within the first 24 h

The patients who had received 100 mcg of ITM had a relatively higher VAS pain score within the first 24 h when compared to those who received 150 mcg ITM after adjusting for the time it took to start feeling pain (Fig. 1). A total of 82.8% patients in the 100 mcg ITM group had pain between 6–15 h that required rescue analgesia, 0 at 18–24 h and 17.2% only developed pain after 24 h. During the period between 18 and 21-h, no patients reported breakthrough pain in this group. In the 150 mcg ITM group, 62.2% patients reported breakthrough pain in the period between 6–15 h, 8.07% between 18–24 h and 29.73% after 24 h. Therefore 150 ITM rendered better analgesic effects than 100 ITM (Table 4). The difference, however, was not statistically significant.

**Table 4:** Comparison of postoperative analgesic rescue between ITM groups

Variable	100 mcg ITM, n (%) 29 (43.94)	≥ 150 mcg ITM, *n* (%) 37 (56.06)	*p*-value
Rescue analgesia	24 (82. 8%)	23 (62.2%)	0.067
No rescue analgesia	5 (17.2%)	14 (37.8%)	

*ITM* intrathecal morphine

**Fig. 2:**
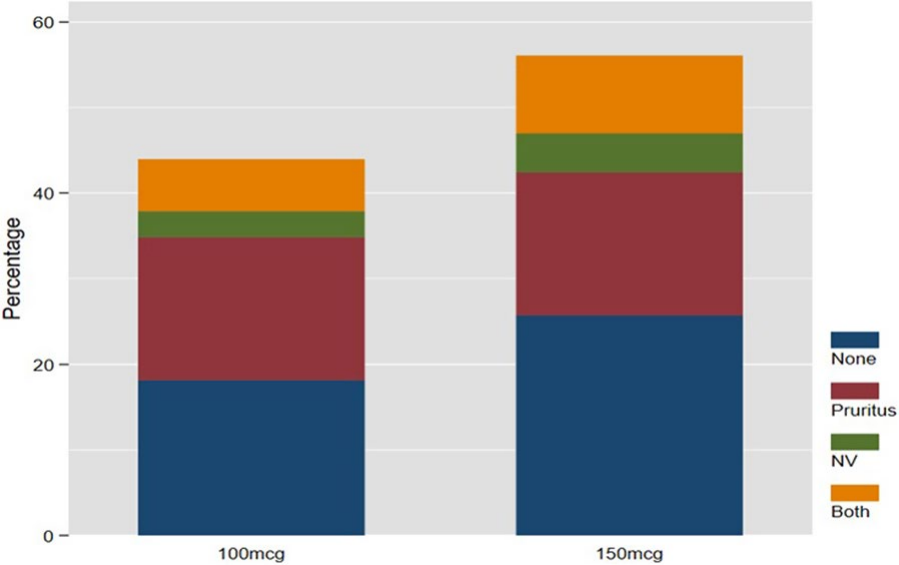
Comparison of side effect profiles between groups

Although the side effect profile was statistically not different (Fig. 2), the percentage of patients who had both pruritus, nausea and vomiting as side effects was high in the 150 mcg ITM group compared to 100 mcg ITM group.

**Fig. 3:**
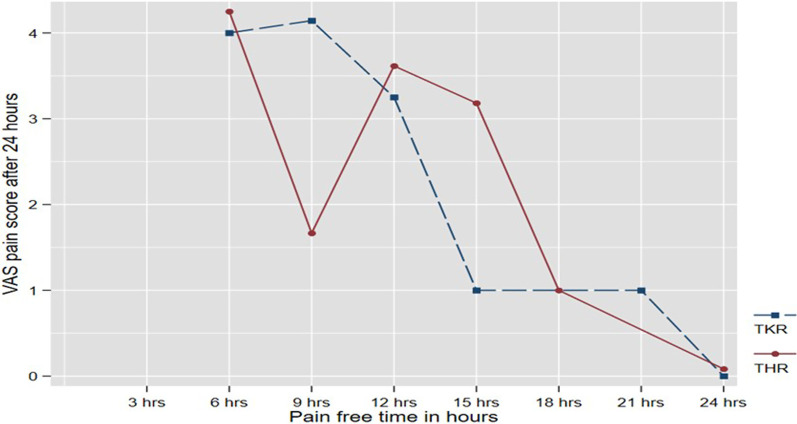
VAS pain score by type of surgery (TKR and THR) adjusted for the pain-free hours

There were no real differences in the VAS pain scores between the two surgeries as observed from the line graphs which cross multiple times in the illustration (Fig. 3).

## Discussion

Median (IQR) VAS pain scores were lower in the 150 mcg ITM group [1 (0–2)] compared to the 100 mcg ITM group [2 (1–5)], *p* = 0.01. Many studies have been done to find the suitable dose of intrathecal morphine or defining the dose of intrathecal morphine in patients undergoing hip and knee arthroplasty which provide good analgesia and less side effects [3, 5, 7, 8, 10, 11, 14–16]. In our setting, the importance is in finding a suitable dose with analgesic effects, and fewer side effects to allow for our patients to be cared for in a normal ward. Dose–response studies demonstrated that ITM has an analgesic efficacy ceiling and the optimal dose of ITM is 0.075–0.15 mg [2].

Although there was no significant difference in pain-free time between the two groups, there was a marginal difference in need for rescue analgesia with those in the 150 mcg ITM needing less. Importantly no cases of respiratory depression were reported, not only making the 150 mcg ITM superior in pain relief but equivalent statistically in side effect profile.

The 150 mcg ITM group had a high percentage of patients who had nausea and vomiting compared to the 100 mcg ITM group. However, the percentage of patients who had both pruritus, nausea and vomiting as side effects was higher in the 150 mcg ITM group compared to 100 mcg ITM group. Previous studies have demonstrated that higher doses of ITM were associated with higher incidences of nausea and vomiting [5, 8, 9, 16]. The incidence and severity of nausea and vomiting is a major concern following the use of ITM particularly as orthopaedic surgery on its own is an independent risk factor of nausea and vomiting [3, 5, 10, 17].

Hassett et al. [7] demonstrated that patients who received 100 mcg ITM had poor pain management compared to the patients who received 200 mcg ITM and 300 mcg ITM. The median time to request first rescue analgesia was lower in 100 mcg IT morphine which was 442 min compare to 200 mcg IT morphine 607 min and 300 mcg IT morphine 857 min. In our study, we reported median time to rescue of 675 min in the 100 mcg ITM group compared to 720 min in the 150 mcg ITM group.

In this study, we found no real differences in the VAS pain scores between the total hip and total knee replacement, *p* = 0.34. A previous study had reported differences in pain scores between these groups [18]. The requirement for rescue analgesia (intravenous morphine) was similarly found to be reduced in patients who have total hip replacement compared to total knee replacement [14].

We found no cases that were reported to have experienced respiratory depression. Some studies use intravenous opioids or patient–controlled analgesia with opioids as rescue analgesia which can also cause respiratory depression [2, 19, 20]. The incidence of respiratory depression has been found to be as low as 3% using a doses 0.2–0.8 mg ITM in one study [21]. We postulate that this may be the contributing factor. Delayed respiratory depression occurs at 6–12 h and may persist up to 24 h [22–24].

The median bupivacaine was 13 mg in the 100 mcg ITM and 14 mg in the 150 mcg ITM group, respectively, and use of phenylephrine and ephedrine were not significantly different, *p* = 0.68 and *p* = 0.46, respectively. Majority of patients were ≥ 50 years old with no differences in baseline characteristics. More had total hip replacement than total knee replacement.

There were no differences in weight between the two groups. We, however, did not anticipate any differences in the pharmacodynamics of the two doses as data on ITM have shown that there is no direct effect from BMI [25]. This is due to its limited volume of distribution which is confined for the most part to the intrathecal space with a volume of distribution 22 ± 8 mL and low protein binding of 36 (25). The analgesic effects of ITM are produced through its effect primarily on spinal mechanisms [26]. It may spread to supraspinal sites due to its relatively high hydrophilicity, and occasionally into systemic circulation [26]. The lowest efficacious dose of morphine is injected the IT space to minimize the risk of respiratory depression [27, 28]. ITM is injected directly into the CSF, close to the structures of the central nervous system which has opioid receptors where morphine acts [29]. In a population of 11,327 women, ITM women with BMI ≥ 40 kg/m^2^ had respiratory adverse events unrelated to neuraxial morphine [30].

## Limitations

The study is contextual with a small sample size. The results cannot therefore be generalized. It, however, presents good data for use in similar circumstances such as low- to medium-income countries with constraint resources.

## Conclusion

A 150 mcg ITM dose can be used with good postoperative analgesic effects, with longer duration of analgesia and reduced postoperative requirement of rescue analgesia compared to 100 mcg in patients scheduled for total hip and knee arthroplasty. Although nausea and vomiting, pruritus were reported at this dose, they were not significantly different and were successfully treated. Most importantly, at this dose, the most dreadful side effect of delayed respiratory depression was not reported. Thus, patients who received 150 mcg ITM were safe in a non-high-dependency area such as a normal ward postoperatively provided anticipated side effects can be monitored and managed timeously.

## Data Availability

The data are available from the corresponding author on request.

## Abbreviations

ITM: Intrathecal morphine
VAS: Visual Analogue Scale
PEP: Phenylephrine
ASA: American society of anaesthesiology
THR: Total hip replacement
TKR: Total knee replacement
NV: Nausea and vomiting
